# Identification of biomarker microRNA-mRNA regulatory pairs for predicting the docetaxel resistance in prostate cancer

**DOI:** 10.7150/jca.29032

**Published:** 2019-08-29

**Authors:** Jian Tu, Qiliang Peng, Yi Shen, Yin Hong, Jiahao Zhu, Zhengyang Feng, Ping Zhou, Shaonan Fan, Yaqun Zhu, Yongsheng Zhang

**Affiliations:** 1 Department of Pathology, The Second Affiliated Hospital of Soochow University, Suzhou, China; 2 Department of Radiotherapy & Oncology, The Second Affiliated Hospital of Soochow University, Suzhou, China; 3 Department of Radiation Oncology, The Affiliated Suzhou Science & Technology Town Hospital of Nanjing Medical University, Suzhou, China; 4 Department of Thoracic Surgery, Suzhou BenQ Hospital, Suzhou, China; 5 Tongda College of Nanjing University of Post and Telecommunications, Yangzhou, China; 6 Department of Oncology, The Second Affiliated Hospital of Soochow University, Suzhou, China

**Keywords:** Docetaxel resistance, Network biomarkers, Bioinformatics analysis, Prostate cancer

## Abstract

**Background**: Docetaxel resistance is a cursing problem with adverse effects on the therapeutic efficacy of prostate cancer (PCa), involving interactions among multiple molecular components. Single or limited molecules are not strong enough as prediction biomarkers of drug resistance. Network biomarkers are considered to outperform individual markers in disease characterization.

**Methods**: In this study, key microRNAs (miRNAs) as biomarkers were identified from the PubMed citations and miRNA expression profiles. Targets of miRNAs were predicted and enriched by biological function analysis. Key target mRNAs of the biomarker miRNAs were screened from protein-protein interaction network and gene expression profiles, respectively. The results were validated by the assessment of their predictive power and system biological analysis.

**Results**: With this approach, we identified 13 miRNAs and 31 target mRNAs with 66 interactions in the constructed network. Integrative functional enrichment analysis and literature exploration further confirmed that the network biomarkers were highly associated with the development of docetaxel resistance.

**Conclusions**: The findings from our results demonstrated that the identified network biomarkers provide a useful tool for predicting the docetaxel resistance and may be helpful for serving as prediction biomarkers and therapeutic targets. However, it is necessary to conduct biological experiments for further investigating their roles in the development of docetaxel resistance.

## Background

Prostate cancer (PCa), a global health problem, is one of the most common cancers and has become the second major cause of cancer death among men 1. Although the prevention and treatment of PCa has made great progress and localized PCa can be effectively treated with less difficulty, the incidence and mortality of this disease remains high as the therapy of aggressive and metastatic forms of PCa is still an ongoing clinical challenge 2, 3. Currently, androgen deprivation therapy is the common treatment of patients with aggressive PCa 4. However, almost all patients will relapse and eventually progress to castration-resistant PCa which will ineluctably lead to cancer metastasis and patients' death 5. Docetaxel is used as the first-line chemotherapeutic agent for castration-resistant PCa and has been determined to show a survival advantage for these patients 6. However, a substantial proportion of men with PCa could not benefit from docetaxel due to the development of drug resistance 7. Therefore, early response prediction of docetaxel before treatment would help prevent potentially nonresponsive patients from unnecessary treatment with possible accompanying side effects and intervene early with sensitization strategies or subject to alternative treatments without delay.

MicroRNAs (miRNAs), which are short non-coding RNAs (18-25 nucleotides), modulate gene expression negatively by binding to messenger RNAs (mRNAs) and prevent them from translating into protein 8. Recently, accumulating evidences have revealed that miRNAs may be involved in the regulation of developmental, physiological and oncogenic processes of various cancers and thus are excellent biomarkers for cancer diagnosis, prognosis and therapeutic efficacy 9, 10. Previously, there have been an increasing number of studies regarding the association of miRNAs with prostate carcinogenesis and the modulation of the clinical course of the disease 11, 12. Meanwhile, several groups of researches have studied the sensitivity to docetaxel could be altered by miRNAs in PCa cells 13. In addition, the development of chemoresistance has been attributed to alterations of the levels of miRNAs 14. Therefore, miRNAs may hold the ability to distinguish responders from non-responders to docetaxel. However, most attention on docetaxel resistance prediction in PCa is addicted to single or limited miRNAs. The clinical applicability of the identified miRNAs as biomarkers of docetaxel resistance of PCa is still limited due to the lack of specificity and sensitivity of individual molecules. As a consequence, there is a great need to develop more integrative and precise methods beyond the traditionally isolated and static mode for miRNA biomarkers discovery.

In this study, we integrated a very wide range of complexly structured data types including miRNA expression information, mRNA expression data, miRNA-mRNA regulatory network, mRNA-mRNA interaction data, and other types of genomic information and combined them into a strong theoretical framework. With several bioinformatics approaches, we aimed to screen the miRNA-mRNA regulatory pairs as network biomarkers for predicting the docetaxel resistance and the potential prediction mechanism underlying these biomarkers. The use of identified miRNAs and their corresponding regulatory mRNAs may also serve as potential therapeutic targets for overcoming docetaxel-resistance for effective PCa treatment.

## Materials and Methods

### Data collection

The previously reported miRNAs related to docetaxel resistance were manually searched in PubMed and only the miRNAs identified by experimental verification or screened by predictive methods were collected. The mRNAs associated with docetaxel resistance were identified from the GenBank database deposited on the public National Center for Biotechnology Information (NCBI) database. The miRNA expression profile (GSE60117) and gene expression datasets (GSE33455, GSE83654) were downloaded from the Gene Expression Omnibus (GEO) based on NCBI 15-17. The miRNA expression profiling was performed on the platform GPL13264 using Agilent-021827 Human miRNA Microarray (V3), which included 21 samples from normal prostate and 56 from prostate cancer. The gene profiling GSE33455 was performed using the platform GPL570 with Affymetrix Human Genome U133 Plus 2.0 Array while GSE83654 was generated by the platform GPL13607 using Agilent-028004 SurePrint G3 Human GE 8x60K Microarray, including docetaxel sensitive and resistant cells.

### Differentially expressed miRNAs (DE-miRNAs) and mRNAs (DE-mRNAs) extraction

The DE-miRNAs between normal prostate and PCa samples and DE-mRNAs between docetaxel sensitive and resistant PCa cells were extracted based on the empirical bayes (eBayes) method in linear models for microarray data analysis (LIMMA) R package 18. For the multiple probes focused on the same index, we selected the probe with most significant variation across its expression profile to calculate the expression. P<0.05 was considered statistically significant.

### Targets of miRNA prediction

The candidate target genes of the identified miRNAs were predicted using three common databases including TargetScan, miRanda and miRDB 19-21. In order to reduce false positive predictions, the miRNA-mRNA regulatory pairs were selected when they existed in all the three databases with three target prediction algorithms, respectively.

### Functional enrichment analysis

To validate the association between the targets of candidate miRNA biomarkers and docetaxel chemoresistance, we carried out an integrative functional enrichment analysis. All the target mRNAs of the identified miRNAs were mapped to the online tool Database for Annotation, Visualization and Integrated Discovery (DAVID) and enriched to perform the Gene ontology (GO) and Kyoto encyclopedia of genes and genomes (KEGG) pathway analyses 22, 23. For evaluating the function of the key DE mRNAs, KEGG pathway and GO enrichment analyses were also conducted with DAVID 24. The p-value < 0.05 was chosen as the cut-off.

### PPI network construction and analysis

In order to assess the interactions among the targets of the identified miRNAs, we retrieved the PPI information by mapping them to the Search Tool for the Retrieval of Interacting Genes (STRING) database and constructed the mRNA-mRNA interaction network 25. The network was then visualized with the powerful tool Cytoscape. Network analysis was carried out to identify the key hub mRNAs with high degrees in the set up network. Module analysis was applied to screen the significant modules active in the network with the plug-in Molecular Complex Detection (MCODE) of Cytoscape. Then functional enrichment was conducted with the selected hub nodes and the genes involved in the screened modules. P<0.05 was set as the cut-off criterion.

## Results

### Detection of candidate biomarker miRNAs for predicting docetaxel resistance

**Figure 1:**
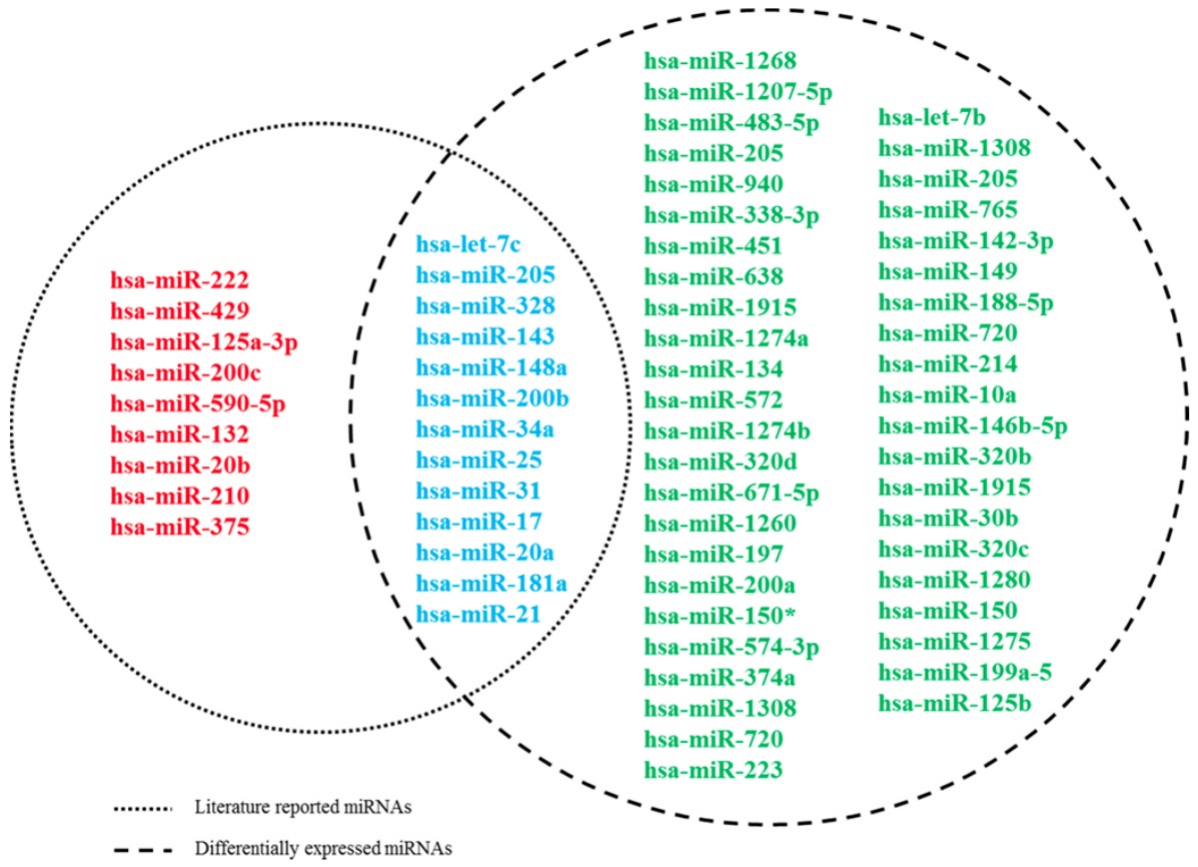
** Venn diagram for literature-reported docetaxel-resistance related miRNAs and DE miRNAs.** Dashed circles on the left and right represent literature-reported miRNAs and DE miRNAs, respectively.

First, by a thorough search in PubMed, a total of 22 literatures reported miRNAs associated with docetaxel chemoresistance were manually collected. All these studies assessed the miRNAs by quantitative real-time PCR (qRT-PCR). Of the 22 miRNAs, 13 were detected in serum of PCa patients, 3 were detected in tissues from PCa patients, the others were measured in cell lines. Furthermore, we used the miRNA expression dataset (GSE60117) to exploit the expression information of these 22 miRNAs. As described in Methods, 13 of the 22 miRNAs were measured differentially expressed between the PCa and normal controls **(Figure 1)**. And finally, these 13 miRNAs were screened as putative miRNA biomarkers for further analysis. The detailed information of the 13 biomarker miRNAs was presented at **Table 1**.

### Identification and characterization of potential target genes of miRNAs

**Figure 2:**
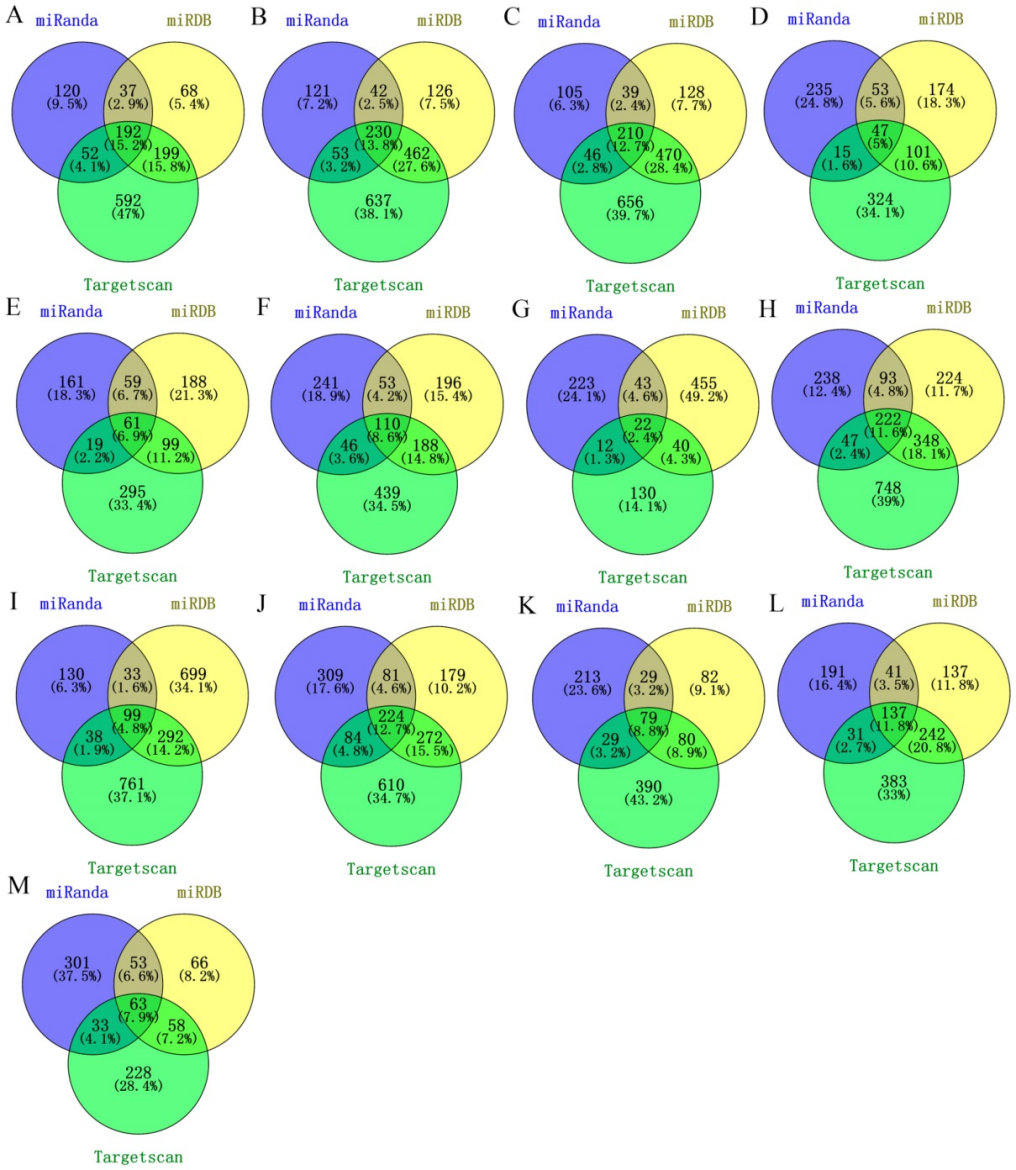
** Targets prediction of identified miRNAs. (A)** miR-25; **(B)** miR-17; **(C)** miR-20a; **(D)** miR-143; **(E)** miR-31; **(F)** miR-34a; **(G)** miR-328; **(H)** miR-181a; **(I)** let-7c; **(J)** miR-200b; **(K)** miR-205; **(L)** miR-148a; **(M)** miR-21

We used three databases (TargetScan, miRanda and miRDB) to predict the target mRNAs of miRNAs by combining predictive and validated miRNA-mRNA pairs. Only the miRNA-mRNA interactions predicted by all the three databases were retrieved (**Figure 2**). As a result, a total of 1296 validated miRNA-mRNA relationship pairs between 13 miRNAs and 1251 mRNAs were simultaneously predicted.

Functional enrichment was performed to make a thorough inquiry for the function of the candidate miRNA biomarkers including GO and KEGG pathway analysis. The miRNA-targeted genes were significantly enriched into a series of GO categories at three different levels including molecular function (MF), cell component (CC) and biological processes (BP). In this study, we mainly concentrated on the top ten significantly enriched terms for in-depth analyses (**Table 2**). At the BP level, most enriched GO terms were mainly linked with the regulation processes including regulation of transcription from RNA polymerase II promoter, actin cytoskeleton organization and cell migration, indicating the regulation function of miRNAs, which are highly associated with the processes of docetaxel resistance. At the CC level, the miRNA targets were most enriched with the hallmarks of a cell including cytoplasm, cytosol, nucleoplasm, which are critical areas with a major impact on the development of chemoresistance. At the MF level, most significant terms were closely relevant to the function of binding such as protein binding, chromatin binding, sequence-specific DNA binding, which also influenced the drug resistance through the binding of important proteins. The GO annotation results indicated the associations between miRNA-targeted mRNAs and docetaxel resistance.

Pathway enrichment analysis further revealed their biological function and the functional mechanisms of the microRNA candidates. A total of 39 pathways were significantly enriched and listed at **Table 3**. Here the top 15 significantly enriched terms were mainly chosen for further literature exploration. The top enriched KEGG terms indicated several pathways related to docetaxel resistance namely pathways in cancer, MAPK signaling pathway, proteoglycans in cancer, adherens junction, TGF-beta signaling pathway, PI3K-Akt signaling pathway and microRNAs in cancer. Based on the above results, we thoroughly screened the enriched functional categories by manually mining citations in PubMed. Pathways in cancer signaling pathway maybe one of the most important pathways as it contains a large number of well-known signaling pathways, such as cell cycle, p53 signaling pathway, MAPK signaling pathway, TGF-β signaling pathway, PI3K-Akt signaling pathway, playing essential roles in cell apoptosis, proliferation, differentiation, invasion and metastasis with high impact on the development of chemoresistance. MAPK signaling pathway, one of the above pathways in cancer, has been inextricably linked to the growth factor-mediated regulation of a variety of biological activities such as cell proliferation, differentiation, migration and chemosensitivity. Recent studies have proposed that activation of MAPK signaling may be responsible for the prostate carcinogenesis and docetaxel resistance 26. As multifunctional molecules, proteoglycans play key roles in diverse cell function during morphogenesis, wound healing, inflammation and tumorigenesis 27. Substantial efforts have convinced that targeting proteoglycans and their modifying enzymes may be helpful for enhancing anticancer chemotherapy efficacy and overcoming drug resistance 28. TGF-β, perhaps the most prominent in the late stages of progression to metastases of PCa, is a bifunctional controller within the tumor microenvironment 29. Accumulating evidence has suggested that disruption of TGF-β signaling may lead to androgen receptor activation and β-catenin nuclear localization, which has been proved to be an adaptation mechanism contributing to emergence of castration-resistant PCa 30. Moreover, TGF-β-targeted therapies may be beneficial for overcoming therapeutic resistance to docetaxel 31. It is well established that PI3K/Akt pathway is closely correlated with cellular survival and cell cycle and particularly regulates the epithelial-to-mesenchymal transition (EMT), an important cellular mechanism in embryonic development, tissue repair, organ fibrosis and cancer metastasis, the role of which in chemoresistance to the therapy and progression of PCa has been investigated by previous studies 32. Aberrant activation of PI3K/Akt pathway may lead to progression of PCa and resistance to apoptosis and inhibition of PI3K/Akt/mTOR pathway has shown anti-tumor effects on docetaxel resistant castration-resistant PCa in vivo and in vitro 33. Recent studies have implied a role of adherens junction in promoting metastasis and chemoresistance by circumventing anoikis and influencing EMT 30. Moreover, the microRNAs in cancer signaling pathway could directly reflect the associations among these miRNAs and their targets in cancer. The pathway analysis revealed the potential mechanism in the development of chemoresistance of docetaxel.

### Integrated network analysis of mRNA-mRNA interactions

**Figure 3:**
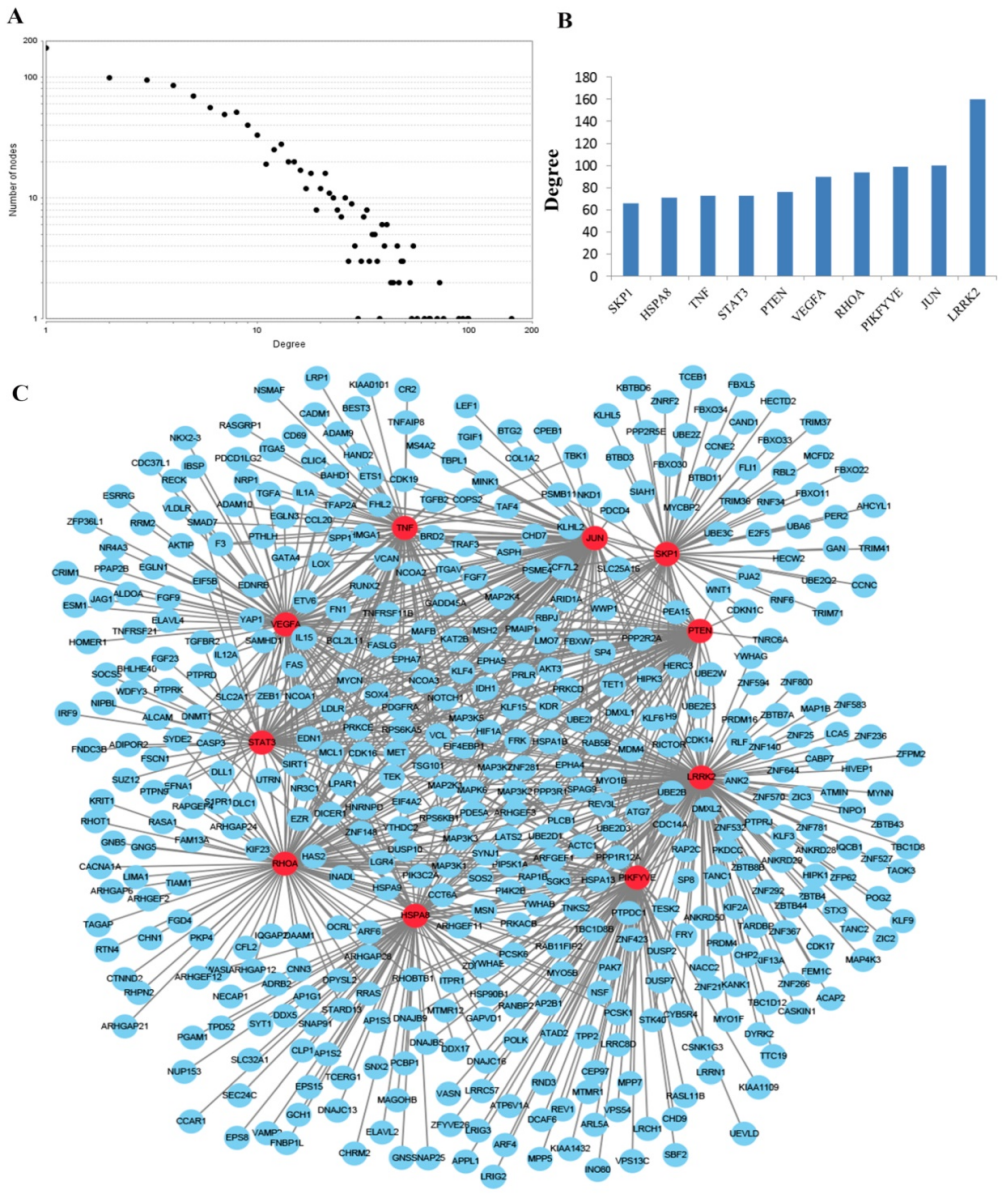
** PPI network construction results. (A)** Degree distributions of nodes for network constructed with all the miRNA targets; **(B)** degree distributions of the top ten hub nodes; **(C)** The sub-network reconstructed with the selected hub nodes and their first neighbor genes. PPI protein-protein interaction

To obtain an improved understanding of the internal contact and interactions among the target mRNAs of the identified miRNAs, the PPI network was set up with the mRNA-mRNA interaction information retrieved from the STRING database. By mapping 1696 mRNAs to STRING, a PPI network was detected and visualized using the Cytoscape platform software made up of 1094 nodes with statistical significance. Network degree reflects the number of interaction partners and the node with the highest degree in the network is indispensable for the stabilization of the network. Therefore, the top 10 hub nodes with higher degrees were screened including LRRK2, JUN, PIKFYVE, RHOA, VEGFA, PTEN, TNF, STAT3, HSPA8 and SKP1. The network analysis results were plotted at **Figure 3**.

**Figure 4:**
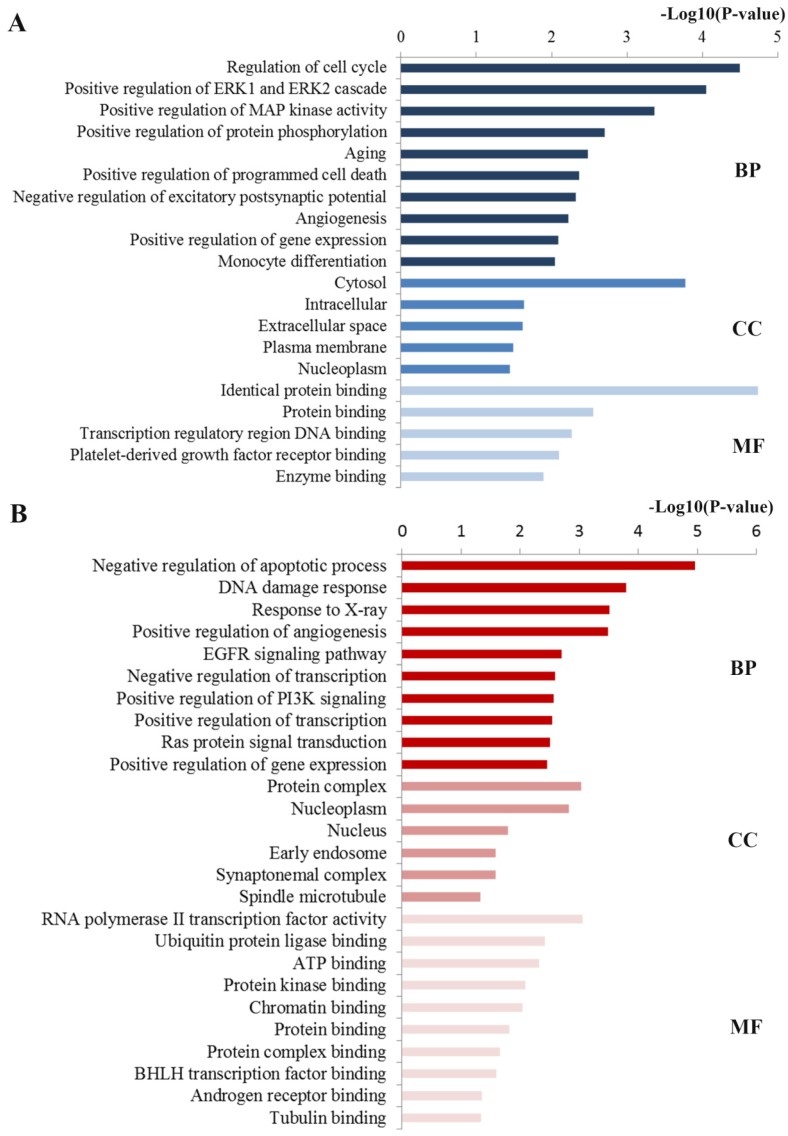
** GO annotation of key miRNA targets. (A)** The significant GO terms enriched by the ten hub mRNAs from the PPI network.** (B)** The significant GO terms enriched by the 21 key DE mRNAs. GO gene ontology, BP biological processes, CC cell component, MF molecular function.

Functional analysis including GO and KEGG pathway analyses were performed to explore the function of these ten key hub nodes. As shown in **Figure 4A**, at the BP level, the most significant terms were closely relevant to regulation processes including regulation of gene expression, excitatory postsynaptic potential and programmed cell death. At the CC level, the enriched terms were mainly concentrated on nucleoplasm, plasma membrane and cytosol. At the MF level, most enriched terms were linked with binding function such as enzyme binding and protein binding. Consistent with the GO analysis of all the targets of the identified miRNAs, these enriched GO results were closely associated with docetaxel resistance.

**Figure 5:**
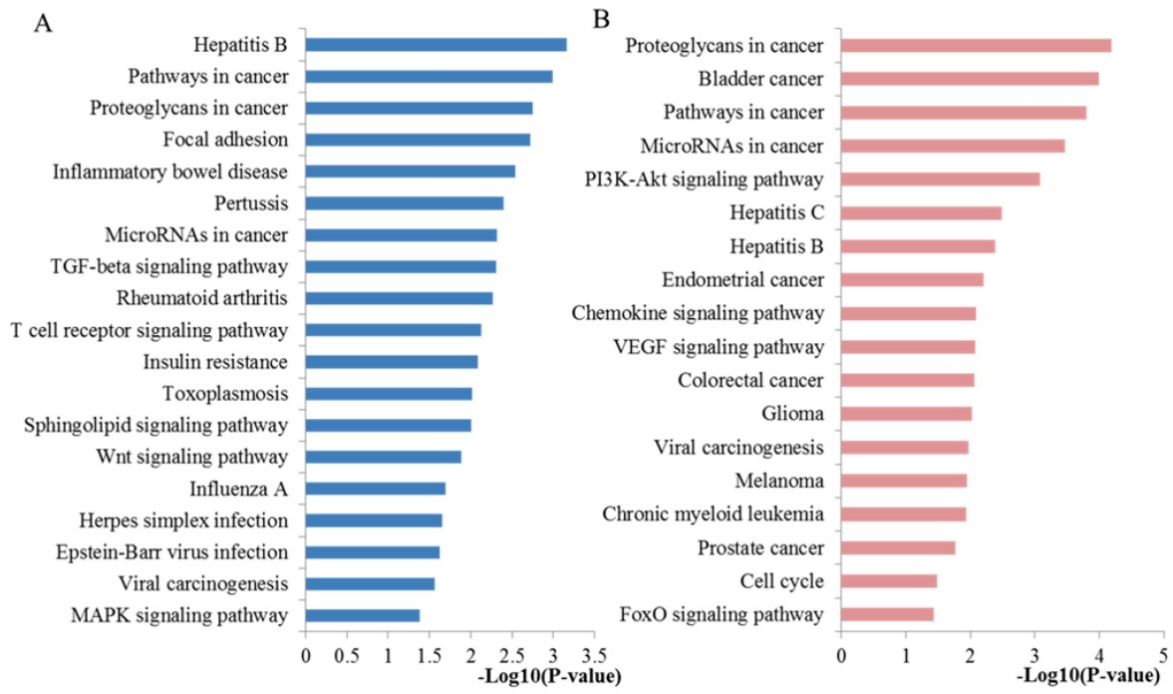
** KEGG pathway enrichment analysis results of key miRNA targets. (A)** The significant pathways enriched by the ten hub mRNAs from the PPI network. **(B)** The significant pathways enriched by the 21 key DE mRNAs.

KEGG pathway analysis was performed to explore the biological function of the identified key hub nodes. All the significant enriched pathways were plotted at **Figure 5A.** The miRNA-targeted genes were significantly enriched into several important pathways including pathways in cancer, proteoglycans in cancer, microRNAs in cancer, TGF-beta signaling pathway, Wnt signaling pathway and MAPK signaling pathway. In addition to most pathways that mentioned above, another part have to mention is that the well-studied Wnt signaling pathway has been a vital pathway mediating prostate tumor microenvironment and chemotherapy sensitivity, indicating that targeting the Wnt pathway could be envisioned as a means to combat this difficult cancer when drug resistant 34, 35.

**Figure 6:**
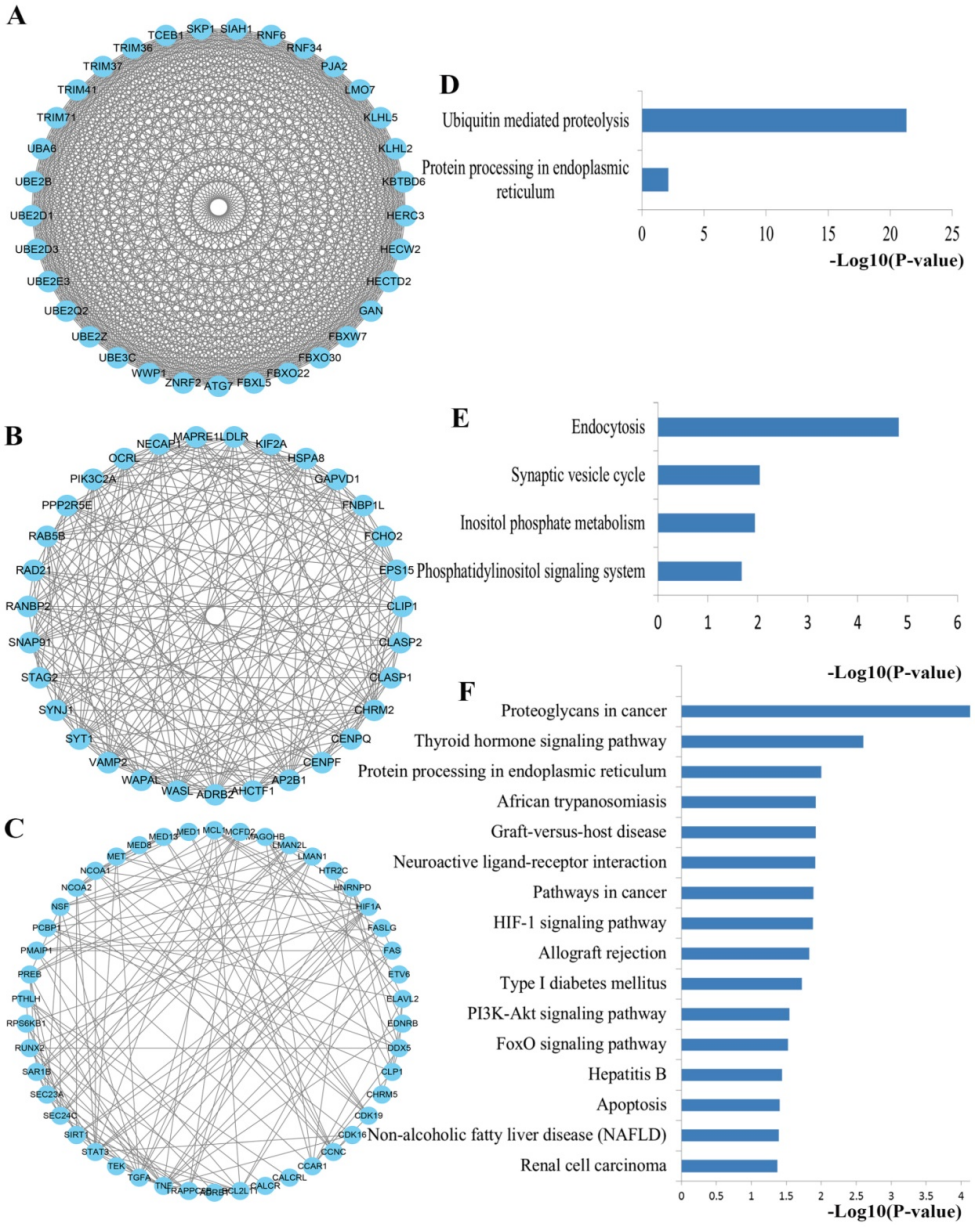
** The top three significant modules from the PPI network. (A-C)** The top three significant modules in the PPI network for miRNA targets; **(D-F)** Pathways enriched by all the nodes involved in the identified three modules, respectively. PPI protein-protein interaction.

Using the MCODE package, the top three significant network modules were obtained from the PPI network (**Figure 6**). Strikingly, all of the genes in the identified modules were significantly enriched into ubiquitin mediated proteolysis, protein processing in endoplasmic reticulum (Module 1), endocytosis, synaptic vesicle cycle, inositol phosphate metabolism, phosphatidylinositol signaling system (Module 2), proteoglycans in cancer, protein processing in endoplasmic reticulum, pathways in cancer, HIF-1 signaling pathway, PI3K-Akt signaling pathway, FoxO signaling pathway and apoptosis (Module 3). In addition to pathways in cancer, PI3K-Akt signaling pathway and FoxO signaling pathway that discussed above, the majority of these pathways are crucial for maintaining intracellular homeostasis under normal conditions with multifunctional roles in PCa cell growth, inflammation, differentiation, apoptosis, and metastasis. For example, ubiquitin regulates the abundance and function of numerous cell proteins, and its inhibition contributes to cancer cell growth inhibition and apoptosis 36. Studies have convinced that apoptosis induced by docetaxel involves some apoptotic signal molecules and the mode of apoptotic action has been identified as vital molecular determinants of resistance and sensitivity to taxanes 37. Furthermore, another part has to mention is that the well-studied HIF-1 signaling pathway has been a vital pathway in the regulation of almost all the enzymes leading to glucose breakdown during glycolysis 38. The activation of HIF-1 may result in the transcription of a plethora of target genes that enhance physiological alterations related to chemoresistance through inhibiting of apoptosis and senescence and activating drug efflux and cellular metabolism. Accordingly, inhibition of HIF-1 might be an attractive strategy to improve the response of the patients with PCa to docetaxel treatment 39. Furthermore, FoxO transcription factors are the central regulator of cellular homeostasis and have various biological functions including cell cycle, cell growth, apoptosis, autophagy, DNA repair, tumor suppression, and metabolism.

Accumulating new evidence supports the concept that altered FoxO signaling contributes to the disruption of microenvironment by promoting cell cycle arrest, apoptosis, stress resistance, and DNA repair in cancer cells and thus results in the chemoresistance 40.

### Screening key miRNA targets and functional analysis

First, mRNAs associated with docetaxel resistance were searched in the GenBank database. A total of 130 mRNAs that may have an important part in the development of the chemoresistance of docetaxel were collected. Next, two gene expression profiles were explored to assess the mRNA expression in the microarray. A total of 1635 and 690 DE mRNAs were obtained respectively in GSE33455 and GSE83654 datasets, of which 20 and 3 were found in the mRNA lists identified from the GenBank database. Interestingly, most of the 23 (21/23) mRNAs were regulated by the identified miRNAs. As a result, we further evaluated the biological function of the 21 key DE mRNAs by mapping them to DAVID.

Based on the GO enrichment analysis (**Figure 4B**), the enriched GO terms in BP mainly included the regulation processes such as regulation of apoptotic process, angiogenesis, transcription and phosphatidylinositol 3-kinase signaling. CC items were associated with the hallmarks of a cell: protein complex, nucleoplasm, nucleus and cytosol. Most GO MF items converged on the binding function such as ubiquitin protein ligase binding, ATP binding, protein kinase binding and chromatin binding. These enriched GO terms were also highly involved in docetaxel resistance.

According to the KEGG pathway analysis (**Figure 5B**), these 21 key DE mRNAs were mainly enriched in pathways like proteoglycans in cancer, pathways in cancer, microRNAs in cancer, PI3K-Akt signaling pathway, chemokine signaling pathway, VEGF signaling pathway, prostate cancer, cell cycle and FoxO signaling pathway. Most of these pathways have been proved associated with docetaxel resistance by literature exploration above. Here, we continued to discuss some more pathways. The well-studied cell cycle pathway, one of the most significant molecular determinants of resistance and sensitivity to cytotoxic agents, has been critically reviewed by a large amount of studies 41. In PCa, deregulations or mutations in the cell cycle regulators may result in the escaping of tumor cells from cell cycle arrest and apoptosis and eventually progress to chemoresistance. Controlling cell cycle may be an attractive strategy for development of PCa therapeutics for overcoming chemoresistance 42. The prostate cancer signaling pathway demonstrated that these miRNA targets were closely associated with the initiation and progression of PCa. VEGF signaling is an endothelial cell specific growth factor specifically involved in mediating developmental angiogenesis and vascular permeability 43. Recent new evidence gathered so far has indicated that VEGF plays a multifunctional role where it can also have autocrine pro-survival effects and lead to tumor cell chemoresistance. Targeting angiogenesis and VEGF receptors has clinically important implications for optimizing the chemotherapy efficacy 44. Recent studies have demonstrated chemokine as an important determinant of cancer cell sensitivity to chemotherapy 45.

### Network biomarkers for predicting the chemoresistance

**Figure 7:**
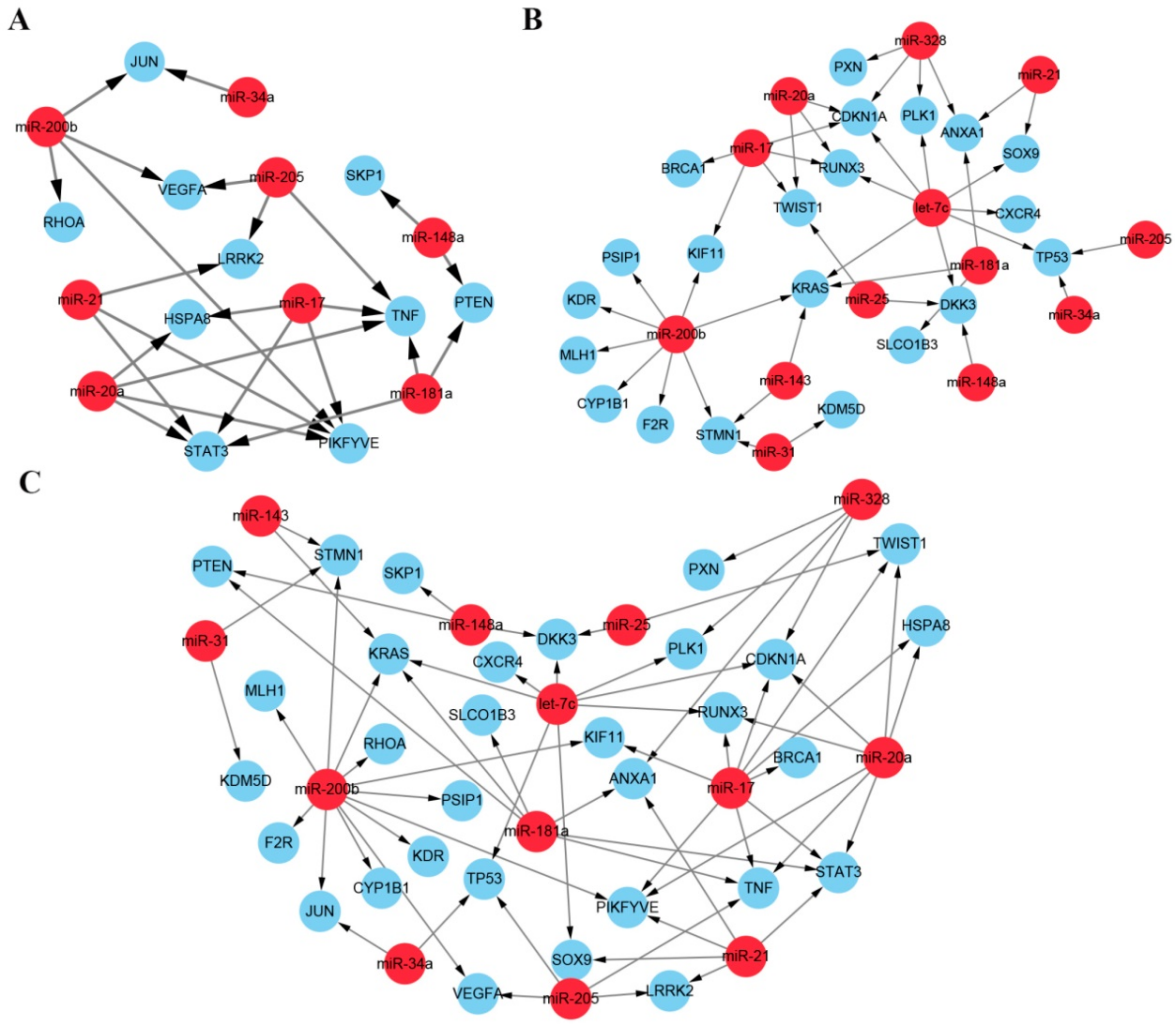
** Network biomarkers for predicting the docetaxel resistance.** (**A**) miRNA-mRNA regulatory pairs from the ten hub mRNAs and their regulated miRNAs. (**B**) miRNA-mRNA regulatory pairs from the DE mRNAs and their regulated miRNAs. (**C**). The whole network set up by the candidate miRNAs and the two lists of mRNAs.

As described above, we screened two lists of miRNA targets, one from the PPI network and another from the mRNA expression profiles. Then we reconstructed the network of the ten hub mRNAs and the 21 DE mRNAs with their regulated miRNAs. In the network, thirteen miRNAs along with 31 target mRNAs constructed the prediction framework including 66 regulatory pairs (**Figure 7**). The identified miRNAs and target mRNAs have been proved highly associated with docetaxel resistance by integrative functional analysis and verification in literatures and may provide potential miRNA-mRNA regulatory pairs as network biomarkers for prediction of docetaxel chemoresistance.

## Discussion

Docetaxel resistance is a cursing problem that prevents the effective treatment of the PCa with adverse effects on the patient's prognosis. Biomarkers for predicting docetaxel chemoresistance may be helpful for better understanding the mechanism underlying the development of resistance and may also provide potential therapeutic targets for overcoming docetaxel-resistance for effective PCa treatment.

An increasingly large amount of evidence has accumulated showing that aberrant expression of miRNAs can contribute to the development of resistant prostate cancers and thus may become perfect biomarkers. In the present study, a total of 22 miRNAs associated with docetaxel resistance in PCa were collected from PubMed with text mining, of which 13 were found differentially expressed in the dataset of GSE60117 containing PCa and normal controls. These 13 miRNAs were identified as candidate biomarkers with high potential involved in docetaxel-resistance as they were supported by relevant researches and could discriminate PCa patients from normal controls.

Recent studies have proposed that miRNAs are involved in the development of resistant prostate cancers through the regulation of their targets. We believe that if the biomarker miRNAs could predict the drug chemoresistance, their target mRNAs may also promote the development of resistant prostate cancers and target multiple signaling pathways related to tumor progression, metastasis, invasion, and chemoresistance. Therefore, an integrated functional analysis was performed with all the regulatory mRNAs of identified miRNAs to explore thoroughly the biological functions of the screened miRNAs and their targets involved in the development of chemoresistance. GO analysis is a common method used for annotating large numbers of genes at the functional level. Most GO terms enriched by the target mRNAs of the identified miRNAs were concentrating on the processes of regulation at the BP level, significantly associated with core cell structural at CC level and mainly including the function of binding at the MF level, which supported the regulatory concepts of miRNAs. Pathway enrichment analysis may provide more precise information in terms of biological functions than GO analysis. With regard to the KEGG pathway enrichment results, the miRNA targets were mainly involved in pathways including proteoglycans in cancer, pathways in cancer, MAPK signaling pathway, adherens junction, TGF-beta signaling pathway and PI3K-Akt signaling pathway. These pathways were all involved in PCa progression and chemoresistance of docetaxel according to previous reports. The functional enrichment analysis agreed well with our exploration of candidate biomarker miRNAs and uncovered the potential mechanisms involved in the drug resistance.

Just as miRNAs and their target mRNAs are closely interrelated, there are also close associations among the miRNA targets. To further investigate the correlations among the target genes of identified miRNAs, we carried out the PPI network analysis. Through PPI network construction, the top ten hub genes were screened. In our study, it was indicated that these key target nodes regulated by the identified miRNAs played important roles in microRNAs in cancer, proteoglycans in cancer, FoxO signaling pathway and MAPK signaling pathway. Moreover, module analysis of the PPI network suggested that the top three significant modules of the identified miRNA targets network were associated with ubiquitin mediated proteolysis, protein processing in endoplasmic reticulum, endocytosis, synaptic vesicle cycle, inositol phosphate metabolism, phosphatidylinositol signaling system, proteoglycans in cancer, protein processing in endoplasmic reticulum, pathways in cancer, HIF-1 signaling pathway, PI3K-Akt signaling pathway, FoxO signaling pathway and apoptosis. We also searched the PubMed literatures for the associations of these pathways and chemoresistance and the results indicated that all the enriched pathways were involved in PCa pathogenesis and docetaxel resistance according to PubMed literature reports.

A single miRNA can regulate multiple target mRNAs. Due to the large number of miRNA targets, we dealt with two mRNA expression datasets to explore their expression in the microarray and identify some key mRNAs. Finally, 21 key miRNA targets were obtained from the selected mRNA expression datasets and supported by the GenBank database that highly associated with docetaxel resistance. According to the KEGG, these 21 key miRNA targets participated in proteoglycans in cancer, pathways in cancer, microRNAs in cancer, PI3K-Akt signaling pathway, chemokine signaling pathway, VEGF signaling pathway, prostate cancer, cell cycle and FoxO signaling pathway. We also conducted the literature search of these pathways to explore their role involved in the docetaxel resistance of PCa.

As described above, two lists of the identified miRNA targets were screened from the PPI network and gene expression datasets, respectively. Although there is no overlapping mRNAs between the two inconsistent lists of mRNA signatures, they become more consistent when mapped to higher functional levels and fall within the similar functional pathways including pathways in cancer, microRNAs in cancer, proteoglycans in cancer and FoxO signaling pathway. In general, functionally associated genes often hold a coordinated expression to execute their roles in the same functional modules, pathways or network, revealing that the two lists of miRNA targets may play a collective role in the drug resistance.

Nowadays, great efforts have been undertaken in search of cancer biomarkers including diagnosis, prognosis and treatment response. However, most attention on treatment response prediction of drug resistance in PCa is addicted to single or limited molecules. As known to all, PCa is a highly complex and heterogeneous disease, the evolutionary process of docetaxel resistance contribute not by the malfunction of single molecules but their synergistic behavior in the network. Therefore, a single biomarker is unlikely to dictate the development of resistance and to say nothing of performing an effective prediction 46. Meanwhile, although microarray analysis allows a full genome display mode simultaneously, bringing enormous convenience for biomarkers discovery, those microarray-based studies have been absorbed in miRNAs or mRNAs separately. Actually, Network biomarkers are considered to outperform individual molecules in disease characterization and response discrimination 47. The network biomarker rooted in systematical and dynamical manner involving miRNA-mRNA interactions could provide novel insights in elucidating the process of drug chemoresistance at the molecular level 48. The network-based approach of the present study integrated a very wide range of complexly structured data types of miRNA and mRNA into a systematical framework and identified docetaxel-resistance associated miRNA-mRNA regulatory pairs as network biomarkers for response prediction. Actually, the ideal method for validating the main findings is to perform biological experiments using two groups of prostate cancer tissue samples from the patients with the same treatment strategy, one from docetaxel chemotherapy sensitive tumor tissues and the other from tumor tissues of docetaxel resistance. However, such tissue samples are difficult for us to obtain. For evaluating the prediction power, although not perfect, an integrative and comprehensive bioinformatics analysis was carried out and our results were successfully verified by recent experimental literatures by text mining. Compared to previous network biomarkers, our miRNA-mRNA regulatory pairs were considered to better characterize the development of drug resistance since all the network elements were closely associated with the chemoresistance of docetaxel. It deserves to be mentioned that when the entire network nodes are integrated into a whole frame, the predictive power may increase. As a future perspective, experimental research verification is warranted when sufficient specimens are available and edge-variation in the network will be considered for developing integrative models with more appropriate and better prediction capacity.

## Conclusion

Taken together, we developed a network approach by integrating a series of complexly structured data into a systematical framework to identify docetaxel resistance associated miRNA-mRNA regulatory pairs as network biomarkers for response prediction. The identification of the most critical miRNAs and their target mRNAs along with the identified pathways correlated well with docetaxel resistance will be the cornerstone for the design of therapeutics. The constructed network biomarkers should be further proved with translational research or clinical studies before they could become clinically useful prediction biomarkers and therapeutic targets.

## Figures and Tables

**Table 1:** Details of candidate miRNA biomarkers

Reported ID	Official ID	P-value (PCa versus Normal controls)	Number of targets
miR-25	hsa-miR-25	4.46E-11	192
miR-17	hsa-miR-17	2.35E-07	230
miR-20a	hsa-miR-20a	6.93E-06	210
miR-143	hsa-miR-143	7.57E-06	47
miR-31	hsa-miR-31	1.72E-04	61
miR-34a	hsa-miR-34a	2.22E-04	110
miR-328	hsa-miR-328	3.17E-04	22
miR-181a	hsa-miR-181a	3.61E-04	222
let-7c	hsa-let-7c	1.06E-03	99
miR-200b	hsa-miR-200b	2.96E-03	224
miR-205	hsa-miR-205	6.24E-03	79
miR-148a	hsa-miR-148a	8.77E-03	137
miR-21	hsa-miR-21	1.16E-02	63

**Table 2:** Top ten significant GO terms enriched by all the targets of the identified microRNA biomarkers

Category	GO terms	Number of enriched genes	P-value
**BP**	Negative regulation of transcription from RNA polymerase II promoter	99	4.44E-11
	Positive regulation of transcription, DNA-templated	78	7.25E-11
	Nervous system development	44	1.13E-06
	Angiogenesis	35	9.96E-06
	Ventricular septum morphogenesis	11	1.37E-05
	Protein transport	51	2.14E-05
	Regulation of actin cytoskeleton organization	13	5.86E-05
	Negative regulation of cell migration	19	7.36E-05
	Negative regulation of stress fiber assembly	8	7.58E-05
	Homophilic cell adhesion via plasma membrane adhesion molecules	26	7.79E-05
**CC**	Cytoplasm	439	6.23E-10
	Cytosol	299	1.58E-09
	Nucleoplasm	250	9.07E-08
	Cell-cell adherens junction	49	9.23E-08
	Focal adhesion	53	9.64E-07
	Transcription factor complex	32	3.38E-06
	Golgi apparatus	92	4.72E-06
	Golgi membrane	68	8.79E-06
	Membrane	194	1.20E-05
	Cytoplasmic vesicle membrane	23	2.31E-05
**MF**	Protein binding	736	6.79E-19
	Ubiquitin-protein transferase activity	47	2.07E-06
	GTPase activator activity	40	1.24E-05
	Cadherin binding involved in cell-cell adhesion	41	1.31E-05
	Chromatin binding	50	2.09E-05
	Sequence-specific DNA binding	61	2.92E-05
	Ubiquitin protein ligase activity	29	5.93E-05
	mRNA 3'-UTR binding	13	7.63E-05
	Transcription regulatory region DNA binding	31	1.04E-04
	Protein kinase activity	44	1.84E-04

**Table 3:** The significantly enriched KEGG pathways by all the targets of candidate miRNA biomarkers

NO.	Pathway term	Number of enriched genes	P-value
1	MAPK signaling pathway	43	7.31E-08
2	Proteoglycans in cancer	33	5.26E-06
3	Pathways in cancer	51	1.29E-05
4	PI3K-Akt signaling pathway	46	1.91E-05
5	Adherens junction	16	7.52E-05
6	Renal cell carcinoma	15	1.04E-04
7	TGF-beta signaling pathway	17	1.61E-04
8	Dorso-ventral axis formation	9	3.30E-04
9	Endocytosis	32	1.56E-03
10	Thyroid hormone signaling pathway	18	1.92E-03
11	MicroRNAs in cancer	34	2.07E-03
12	Ubiquitin mediated proteolysis	20	2.53E-03
13	Axon guidance	19	2.56E-03
14	Regulation of actin cytoskeleton	27	2.57E-03
15	Colorectal cancer	12	3.02E-03
16	Ras signaling pathway	28	3.34E-03
17	Focal adhesion	26	3.79E-03
18	Rap1 signaling pathway	26	4.88E-03
19	Wnt signaling pathway	19	6.28E-03
20	HIF-1 signaling pathway	15	6.89E-03
21	Neurotrophin signaling pathway	17	7.87E-03
22	FoxO signaling pathway	18	1.02E-02
23	Synaptic vesicle cycle	11	1.03E-02
24	Fc gamma R-mediated phagocytosis	13	1.19E-02
25	Amoebiasis	15	1.35E-02
26	Hippo signaling pathway	19	1.54E-02
27	Oocyte meiosis	15	1.70E-02
28	Sphingolipid signaling pathway	16	1.73E-02
29	GnRH signaling pathway	13	2.15E-02
30	TNF signaling pathway	14	2.95E-02
31	Central carbon metabolism in cancer	10	3.01E-02
32	GABAergic synapse	12	3.07E-02
33	ECM-receptor interaction	12	3.56E-02
34	Acute myeloid leukemia	9	3.66E-02
35	RNA degradation	11	3.75E-02
36	p53 signaling pathway	10	3.91E-02
37	AMPK signaling pathway	15	4.03E-02
38	Melanogenesis	13	4.12E-02
39	Retrograde endocannabinoid signaling	13	4.40E-02

## Abbreviations

PCa: prostate cancer
GO: gene ontology
KEGG: Kyoto Encyclopedia of Genes and Genomes
PPI: protein-protein interaction
DAVID: Database for Annotation, Visualization, and Integrated Discovery
STRING: Search Tool for the Retrieval of Interacting Genes
MCODE: Molecular Complex Detection
BP: biological process
CC: cellular component
MF: molecular function
GEO: Gene Expression Omnibus
NCBI: National Center for Biotechnology Information
DE: Differentially expressed
